# Comparative structural analysis of haemagglutinin proteins from type A influenza viruses: conserved and variable features

**DOI:** 10.1186/s12859-014-0363-5

**Published:** 2014-12-10

**Authors:** Irene Righetto, Adelaide Milani, Giovanni Cattoli, Francesco Filippini

**Affiliations:** Molecular Biology and Bioinformatics Unit (MOLBINFO), Department of Biology, University of Padua, via U. Bassi 58/B, 35131 Padova, Italy; FAO-OIE and National Reference Laboratory for Newcastle Disease and Avian Influenza, Istituto Zooprofilattico delle Venezie (IZSVe), viale dell’Università 10, 35020 Legnaro, Italy

**Keywords:** Haemagglutinin, Avian influenza virus, Viral evolution, H5N1, Antigenic drift, Receptor binding domain, Homology modeling, Isopotential contour, Hydropathy analysis

## Abstract

**Background:**

Genome variation is very high in influenza A viruses. However, viral evolution and spreading is strongly influenced by immunogenic features and capacity to bind host cells, depending in turn on the two major capsidic proteins. Therefore, such viruses are classified based on haemagglutinin and neuraminidase types, e.g. H5N1. Current analyses of viral evolution are based on serological and primary sequence comparison; however, comparative structural analysis of capsidic proteins can provide functional insights on surface regions possibly crucial to antigenicity and cell binding.

**Results:**

We performed extensive structural comparison of influenza virus haemagglutinins and of their domains and subregions to investigate type- and/or domain-specific variation. We found that structural closeness and primary sequence similarity are not always tightly related; moreover, type-specific features could be inferred when comparing surface properties of haemagglutinin subregions, monomers and trimers, in terms of electrostatics and hydropathy. Focusing on H5N1, we found that variation at the receptor binding domain surface intriguingly relates to branching of still circulating clades from those ones that are no longer circulating.

**Conclusions:**

Evidence from this work suggests that integrating phylogenetic and serological analyses by extensive structural comparison can help in understanding the ‘functional evolution’ of viral surface determinants. In particular, variation in electrostatic and hydropathy patches can provide molecular evolution markers: intriguing surface charge redistribution characterizing the haemagglutinin receptor binding domains from circulating H5N1 clades 2 and 7 might have contributed to antigenic escape hence to their evolutionary success and spreading.

**Electronic supplementary material:**

The online version of this article (doi:10.1186/s12859-014-0363-5) contains supplementary material, which is available to authorized users.

## Background

Influenza caused by influenza A viruses occurs in both birds and mammals. In humans, influenza A viruses infect hundreds of millions individuals, causing a high number of deaths per year. Indeed, influenza A outbreaks occurred in 1918, 1957 and 1968 resulted in death for ~100 million people worldwide [1]. However, seasonal epidemic outbreaks cause estimated 250.000 to 500.000 yearly deaths worldwide [2] (data from the World Health Organization (WHO) [3] and from the Center for Disease Control and prevention [4]). The largest reservoir of all subtypes of influenza A is found in wild water avian species and some viruses can infect different hosts [5,6]. Classification of influenza type A virus subtypes is based on antigenic and genetic differences in the two surface spike proteins: haemagglutinin (HA) and neuraminidase. For instance, H5N1 viruses combine the haemagglutinin of the H5 subtype with neuraminidase of the N1 subtype. A wide interest for haemagglutinin depends on evidence that this protein (i) is crucial to the attachment and penetration into the host cell, (ii) represents the main viral surface antigen, and (iii) is a major player in the stimulation of the neutralizing antibody response [7]. Haemagglutinin is synthesized as a precursor and then processed by cellular proteases to yield mature polypeptide subregions. In order to provide unambiguos information, hereafter acronyms for haemagglutinin are the followings: ‘HA’ for haemagglutinin in general; HA0 for the precursor; HA1 and HA2 for the two subregions and ‘H’ followed by progressive numbering (H1 to H16) for each haemagglutinin subtype. Influenza virus haemagglutinin is a type I transmembrane glycoprotein that is exposed at the viral surface as a homotrimer. Trimerization is possible once proteolytic cleavage of the unfolded HA0 precursor occurs hence allows for folding of monomers, each consisting of two mature chains: HA1 and HA2 [7]. Structurally, each monomer consists of a globular ‘head’ (part of chain HA1) and of a ‘stem’ region (contributed by both chains HA1 and HA2). The head includes a receptor-binding domain (RBD) and a vestigial esterase domain (VED), whereas the stem is structured as a mainly α helical, coiled coil region. Functionally, the RBD mediates docking to the host cell by binding sialic acids as cell entry receptors, whereas the stem domain mediates both tethering and membrane fusion once conformational change is occurred, caused by pH decreasing along the endosomal route. For several years, classification of HA from influenza viruses was mainly based upon serological and/or phylogenetic analysis [8]. However, structural genomics projects are providing the scientific community with an increasing number of structural templates, while contemporary reverse genetics, immunogenomics investigations and improved sequencing technologies are producing a high number of mutant sequences. Changes in serological specificity depend on variation of epitopes recognized by the specific antibody rather than on the extent of sequence divergence, meaning i.e. that (i) two proteins with highly similar sequences may show quite different properties when considering recognition of specific epitopes and (ii) two proteins may share antigenic properties even when having highly divergent sequences, if epitopes involved in the specific recognition were conserved. Variation of some protein properties sometimes may depend only on ‘local and limited changes’, e.g. mutation of a few - or even only one – residue(s) within linear or conformational motifs. In fact, even when local variation in sequence is seemingly poorly evident, it may result in ‘locally dramatic’ changes in accessible surface area, electrostatic potential, hydropathy or hydrophilicity features that can deeply change motif functionality. It is common knowledge that variation in surface features of a protein can modulate ‘recognition’ interactions of the protein itself. Since variation often depends on mutation of a number of residues and changes in side chains can vary multiple biochemical features, it is difficult or even nonsense trying to establish *a priori* which specific property (among e.g. surface area and shape, electrostatics or hydrophobicity) should be more relevant than others in modulating recognition interactions. In fact, changes in each specific property can result in such modulation, and this can be independent on variation of other features, or modulation can result from the aggregate or synergistic effect of multiple feature changes. So far, several sequence-based studies on variation could provide valuable phylogenetic evidence; however, such studies are of minor help in inferring variation at protein regions including amino acids that are far each other in the primary sequence and quite close within the 3D protein structure (conformational epitopes). In practice, while sequence-based investigation can be good in highlighting very evident changes at individual positions of a protein chain, in general they fail in highlighting meaningful ‘group variation’, i.e. in identifying - especially when the overall variation is relevant and spread - relationship of specific multiple changes to variation in conformational epitopes hence in interactions they mediate.

Once solved structures are available, presence of one or more structural templates allows for shifting to ‘conformational epitope based’ studies on variation and, in particular, to investigating on surface region variation. Stressing relevance of local surface variation is particularly important when considering special constraints addressing viruses evolution: keeping basic properties in simplified but complex pathogenic systems while simultaneously varying - as much as possible - all variable epitopes, in order to escape the immune responses of their hosts. Therefore, viral genome evolution runs along two parallel tracks, both of which, like in railways, must be followed: (i) mutations in sites crucial to protein machinery mediating basic functions (e.g. in motifs relevant to host recognition or cell entrance) are not allowed because they strongly impair viral fitness, and at the same time, (ii) hyper-variability is needed to escape recognition by neutralizing antibodies (‘antigenic drift’, [7]). Given that surface viral proteins do not interact only with antibodies (as their original function is to contact the host), in addition to determining antigenic drift, variation can also influence pathogenicity (because e.g. of modified interaction with cell receptors in different tissues and organ districts) or host specificity. Influenza viruses do not escape such a two-tracks rule, hence while global structure conservation ensures basic functions, limited or even subtle changes in local structural features may modulate interactions of the viral proteins with the host molecules/cells and thus mechanisms underlying antigenic drift, pathogenicity shifts and host specificity change. Phylogenetically and serologically, haemagglutinins are divided into either two supergroups or four groups: Group 1 (H1, 2, 5, 6, 11, 13 and 16); Group 2 (H8, 9 and 12); Group 3 (H3, 4 and 14) and Group 4 (H7, 10 and 15). The two supergroups consist of Groups 1 + 2 and 3 + 4, respectively [9,10]. Thanks to the availability of thousands of viral genomes/gene sequences and of several specific antibodies/vaccines, a large number of sequence-based/phylogenetic and serological analyses of avian flu viruses have been performed and published so far. This notwithstanding, mechanisms in viral evolution are still elusive, as genome/proteome-wide analyses on sequence variation or antigenic features are able to only partially unveil a number of relevant changes, because of the overall mutational noise. Therefore, structural ‘zoom in’ is needed to integrate such analyses by identifying ‘meaningful’ variation. This prompted us to take advantage from availability of structural templates to perform structural comparison among different HA subtypes, in order to identify subtype- and subregion-specific feature variation suggestive for possible involvement in antigenic recognition, or pathogenicity and host specificity. Last but not least, evidence from structural comparison can check relationship among serological, phylogenetic and structural closeness.

We started our analyses using six currently available solved HA structures; then, in order to investigate structural variation possibly underlying H5N1 clades evolution and spreading, we also created clade models by homology modeling. The six HA structures solved so far: H1 [11], H2 [12], H3 [13], H5 [14], H7 [9], H9 [15], all concern mature proteins, consisting of the two HA1 and HA2 parts of haemagglutinin. Solved structure of H16 [16] was not considered for this analysis because it corresponds to the HA0 precursor. Comparative analysis of structural features unveiled that some discrepancy may occur with respect to a generally observed agreement between sequence and structural closeness, because of subregion local variation. Structural analysis was performed by comparison of secondary structure topology and surface analysis, in terms of both electrostatic and hydropathy analysis.

## Results and discussion

### Comparison among solved HA structures

Prior to creating models, preliminary analysis of the six available HA structures was performed in order to evaluate intra- and inter-group structural variation by superposition of all structure pairs and computation of their Root Mean Square Deviation (RMSD). Indeed, the RMSD of two superposed structures indicates their ‘structural divergence’ from one another. As both sequence mutation and conformational variation inflate the RMSD, values up to 2 Ångstrom indicate structural similarity [17]. Structural superposition of each possible combination of two different HA molecules (hereafter referred to as ‘pairs’) and RMSD computing were performed using Chimera 1.8.1 software [18]. Pair-wise method was chosen to calculate RMSD because all superpositions only compared pairs in order to properly relate a structural closeness index for a pair to identity/similarity values (commonly reported as an index to state closeness) from the corresponding aligned sequences. Fold comparison method based on sequence fragmentation and order-independent resorting was not considered because order-dependent global alignment is an established standard for comparing highly similar sequences in structural biology and the alignment of sequence blocks for phylogenetic analyses is also order-dependent.

In addition to superposing structures of HA monomers, also corresponding structures of their Receptor Binding domains (RBDs) were superposed. Results are summarized in Table 1. Evidence that RMSD values for monomer pairs are lower than those ones for corresponding HA1 or RBD regions is not surprising, because RBDs are major determinants in antigenic variation [9]. Moreover, HA2 ‘stem’ region of the monomer is structurally less variable than HA1 [19], hence its contribution results in decreasing the overall monomer RMSD value. RMSD values for HA1 pairs are higher than corresponding RBDs because of unstructured regions connecting RBDs to stems. Group 1 is - at least to date - the only HA group in which multiple structures (in particular, H1, H2 and H5) are solved. Structural comparison within this group highlights some intriguing evidence. When comparing monomers amino acid sequences, H5 results to be closer to H2 than to H1, independently on identity (roughly 73% vs. 63%) or similarity (approximately 86% vs. 81%) is considered. Such relationship is confirmed for both HA1 and RBD sequences, as shown by identity and similarity values in Table 1. However, when comparing structures, H5 is closer to H1 than H2, as in all comparisons, H5:H1 superposition RMSD values are lower than H5:H2 ones. Commonly, % identity is taken into account as an index for relationship among proteins [20]. However, from a structural point of view, ‘type’ of mutations occurred - rather than the overall sequence divergence - is very important: a few mutations (or even a single one) to some specific residues in ‘critical’ regions can result in dramatic structural changes. Structural fold and architecture can be highly conserved even among proteins and protein domains showing no sequence homology because of either long evolutionary divergence or even convergent evolution [21]. At the same time, within such families, fold can be disrupted (resulting in loss of function and disease) by single or few specific mutation(s), which indeed result in keeping 99% or higher sequence identity values [22,23]. In the structural comparison of H5 to haemagglutinins from different groups (represented by H9, H3 and H7) further interesting points emerge. In the monomer comparison, % identity approximately ranges from 41 to 49%. The same 8% difference in % identity is retrieved in % similarity (ranging from 64 to 72%). However, RMSD for corresponding monomer pairs keep quite similar values, i.e. they are not impaired by lower % identity or similarity values. This is not surprising, because - as shown by aforementioned example (and by many others in literature) - very ancient divergence or convergence can result in fold conservation among proteins without significant sequence similarity. Structural differences become clearly evident when comparison focuses on HA1 and RBD regions: H5 is quite closer to H9 than H3 and H7 (roughly doubled RMSD) and in this instance substantial agreement between structural and sequence divergence is found. Once again, a rationale for this is found when considering common properties of protein domains. Different subregions of the same protein are involved in different interactions and pathways. Therefore, molecular evolution can locally change subregion structures to modulate specific interactions and pathways, without affecting those ones mediated from other subregions of the same protein. In practice, only when structural variation analysis is performed at both overall and local level (i.e. focusing on individual domains and/or domain motifs), it is possible to boost subsequent experimental work. In fact, subregion analysis allows for shedding light on specific molecular properties that are likely to underlie different functions of the protein. In conclusion, agreement between sequence homology and structural closeness which is generally observed [20] has not to be strictly interpreted as ‘a rule’ to be followed. Values from Table 1 show that, in most instances, such an agreement is found. However, in several examples and depending on local variation, superimpositions between pairs with quite comparable % identity and similarity may show very different RMSD values and vice versa.

**Table 1:** **Structural and sequence closeness among pairs of haemagglutinin proteins with solved structures**

	**RBD**				
	**H2**	**H5**	**H9**	**H3**	**H7**
**H1**	r:1.343	r:0.918	r:1.249	r:2.292	r:2.784
	i:55.4 s:78.4	i:52.0 s:78.3	i:45.7 s:69.7	i:38.0 s:61.1	i:37.2 s:63.7
**H2**		r:1.130	r:1.636	r:2.083	r:1.772
		i:65.6 s:83.7	i:41.4 s:66.8	i:36.8 s:57.3	i:33.5 s:60.7
**H5**			r:1.498	r:2.241	r:3.085
			i:41.4 s:66.4	i:37.3 s:61.4	i:38.4 s:67.4
**H9**				r:1.983	r:2.069
				i:36.9 s:60.4	i:33.9 s:58.4
**H3**					r:1.429
					i:35.0 s:63.6
	**HA1**				
	**H2**	**H5**	**H9**	**H3**	**H7**
**H1**	r:1.476	r:1.065	r:1.563	r:2.548	r:2.941
	i:56.7 s:78.7	i:56.6 s:79.2	i:46.4 s:69.4	i:37.1 s:62.9	i:36.1 s:63.3
**H2**		r:1.527	r:2.087	r:3.253	r:3.025
		i:67.7 s:83.3	i:43.5 s:65.3	i:35.3 s:58.3	i:34.5 s:60.6
**H5**			r:1.680	r:3.043	r:2.755
			i:43.5 s:67.0	i:37.2 s:61.9	i:36.9 s:66.7
**H9**				r:2.320	r:3.672
				i:35.8 s:60.9	i:33.5 s:59.8
**H3**					r:1.631
					i:37.8 s:64.0
	**Monomer**				
	**H2**	**H5**	**H9**	**H3**	**H7**
**H1**	r:1.180	r:0.98	r:1.350	r:1.710	r:1.780
	i:64.2 s:82.9	i:62.8 s:81.5	i:50.4 s:71.3	i:40.0 s:61.6	i:42.4 s:67.1
**H2**		r:1.100	r:1.450	r:1.760	r:1.730
		i:73.0 s:85.7	i:49.0 s:69.6	i:37.6 s:59.6	i:40.6 s:66.5
**H5**			r:1.686	r:1.680	r:1.620
			i:48.7 s:72.0	i:40.2 s:63.9	i:42.3 s:69.9
**H9**				r:1.760	r:1.850
				i:37.9 s:61.7	i:40.8 s:66.1
**H3**					r:1.250
					i:44.0 s:66.2

Within each cell, the upper value is RMSD (r) for the superposed pair and lower values (in %) are identity (i) and similarity (s) for corresponding, aligned amino acid sequences.

### Comparative analysis of secondary structure elements

Available structures were superposed and then tiled using UCSF Chimera 1.8.1 to keep the same orientation and to avoid visual superposition. This way, variation of secondary structure elements among individual structures can be clearly distinguished and viewed. In order to exclude any artifact from modeling, only the six available solved structures were compared. In terms of secondary structure, three subregions can be distinguished within the HA2 stem [see Additional file 1, panel A]: an α subregion and two β subregions (being either proximal or distal to the VED). The former consists of α helices A-C-D and the B loop (that upon fusion becomes B helix [1]). No meaningful variation - in terms of secondary structure - is found in the α subregion of the stem, because structural changes only concern the B loop [see Additional file 1, panel B], which indeed is unfolded in the pre-fusion state. The B loop coordinates depend on crystallization conditions and in particular on pH [14]. The VED-proximal and distal β subregions are recognized by respectively antibodies CR6261 and CR8020 [24]. The VED-proximal β subregion shows a varying number (zero, two or four) of β strands [see Additional file 1, panel C] and such variation is not relevant to antibody recognition specificity. For instance, a four-strands structure is shared between H5 (recognized by CR6261) and H3 (not recognized); moreover, a two-strands structure is shared between H2 (recognized) and H7 (not recognized). Secondary structure variation is evident also in the distal β subregion [see Additional file 1, panel D], but once again it does not relate to antibody recognition: e.g., CR8020 recognizes subregion from H7 but not corresponding one from H5. Given that subregions recognized by each antibody are clearly different (CR6261 recognizes H1, H2, H5 and H9 independently on they are showing either zero, two or four β strands) such a preliminary analysis demonstrates that secondary structure variation as viewed by cartoon representation is not indicative for epitope variation. Secondary structure variation in the globular RBD-VED region is poorly evident, according to the aforementioned ‘two-tracks’ rule: mutations altering the overall backbone/fold of the RBD would impair binding to host cells hence conservation (track 1) is needed to keep such basic function. However, local variation (track 2) is needed to modulate surface features hence interactions. Therefore, we did not further investigate secondary structure variation and moved instead to surface analysis, considering both most relevant features: (i) electrostatic charge distribution and (ii) hydropathy/hydrophilicity patches.

### Comparative analysis of electrostatic potentials

**Figure 1:**
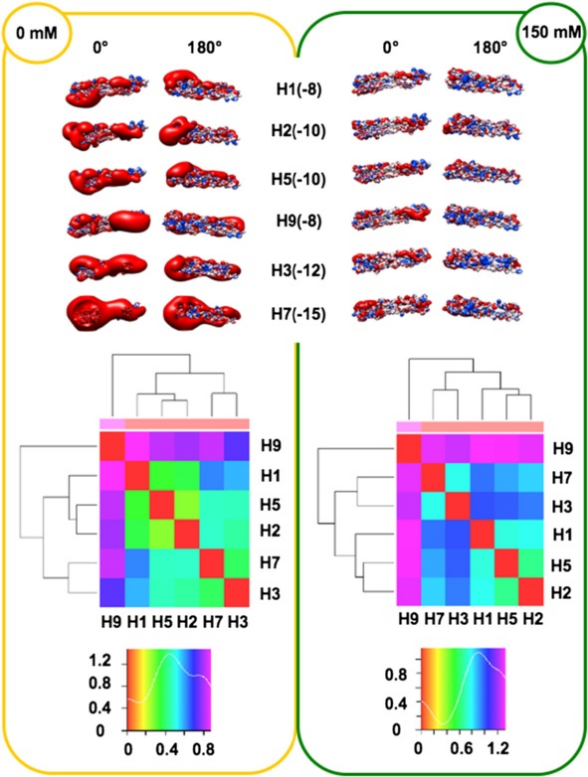
**Isopotential contours (top), heat maps (middle) and density plots (bottom) of HA stems.** Electrostatic features are shown at I = 0 mM (left panel, yellow) and I = 150 mM (right panel, green). Electrostatic potentials (blue for positive and red for negative) are presented in two orientations (0° and 180°). Isopotential contours are plotted at ±3k_B_T/e. In heat maps, red, warm and cold colors correspond to identity, low and high Electrostatic Distances (ED), respectively. The number of hits (pairs of compared structures) is plotted and referred to as ‘Density’.

**Figure 2:**
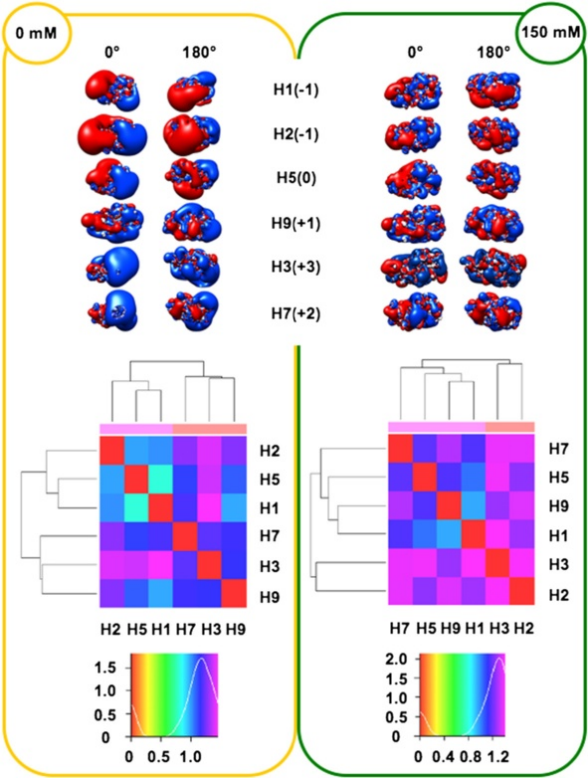
**Isopotential contours (top), heat maps (middle) and density plots (bottom) of HA RBDs.** See Figure 1 caption for color code and definitions. Isopotential contours are plotted at ±1k_B_T/e.

**Figure 3:**
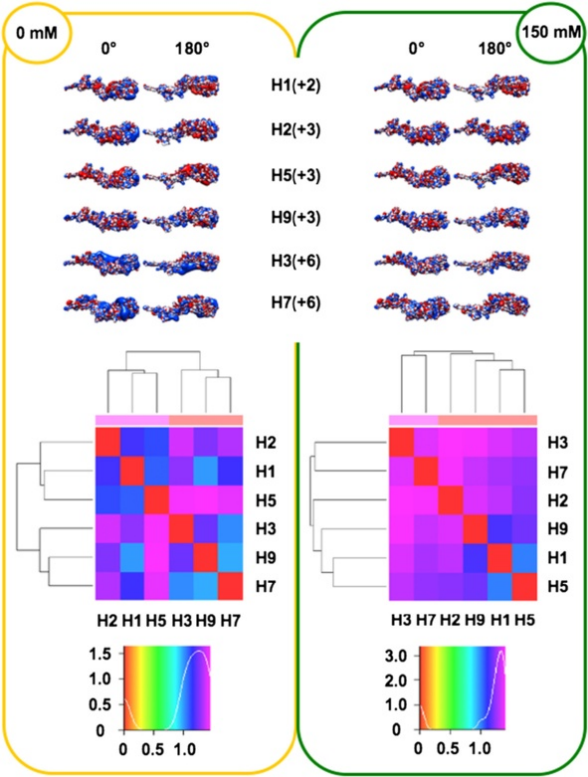
**Isopotential contours (top), heat maps (middle) and density plots (bottom) of HA1 subregions.** See Figure 1 caption for details. Isopotential contours are plotted at ±2k_B_T/e.

**Figure 4:**
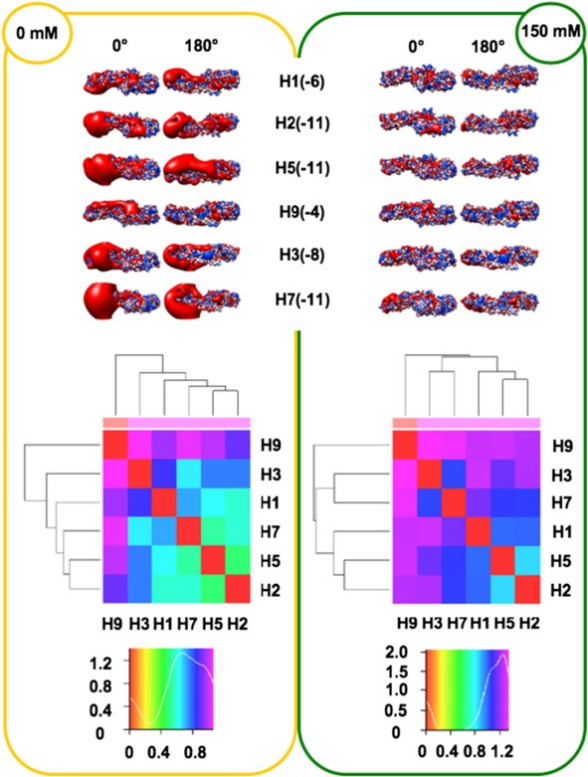
**Isopotential contours (top), heat maps (middle) and density plots (bottom) of HA monomers.** See Figure 1 caption for details. Isopotential contours are plotted at ±2k_B_T/e.

**Figure 5:**
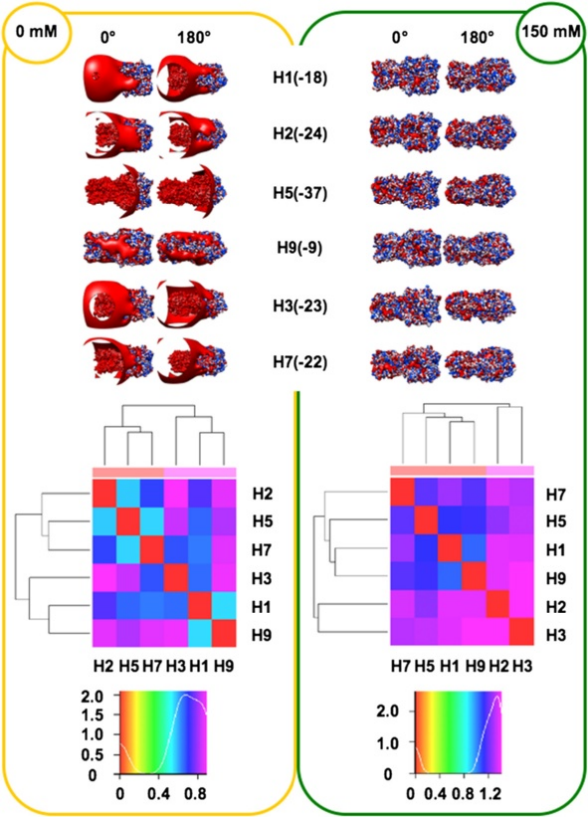
**Isopotential contours (top), heat maps (middle) and density plots (bottom) of HA trimers.** See Figure 1 caption for details. Isopotential contours are plotted at ±3k_B_T/e.

In order to perform analyses taking into account the influence of ionic strength (I), the spatial distribution of the electrostatic potential was calculated at both I = 0 mM (Coulombic interactions unscreened by counter-ions) and I = 150 mM (physiological), assuming +1/-1 charges for the counter-ions. Prior to electrostatic potential calculations, partial charges and van der Waals radii were assigned with PDB2PQR [25,26]; then, linear Poisson-Boltzmann (PB) equation calculations were carried out by using Adaptive PB Solver (APBS) [27] through Opal web service (see Methods). The spatial distribution of the electrostatic potential was determined for each HA subregion, monomers and trimers, comparing the six available HA structures to identify possible HA-specific signatures. In particular, we focused on the role of charge distribution as visualized by isopotential contours within the tertiary structure and on classifying conservation and divergence among the different HAs. In order to evaluate electrostatic distance (ED) also in a quantitative way, clustering of the spatial distributions of the electrostatic potentials was obtained by WebPIPSA (Protein Interaction Property Similarity Analysis; [28], having the use of Hodgkin and Carbo similarity index (SI) [29] (see Methods). The Carbo SI is sensitive to the shape of the potential being considered but not the magnitude, whereas the Hodgkin SI is sensitive to both shape and magnitude. Therefore, WebPIPSA results obtained using the Hodgkin SI are shown in Figures 1, 2, 3, 4 and 5, and evidence from analyses performed using the Carbo SI is cited to confirm parameter independent data.

#### Stem subregions

The electrostatic patches at ionic strength I = 0 mM clearly show for all six stems preferential side disposition (Figure 1, top left), as observed for SNAREs [30]. In particular, density of negative potential (red) at the 0° side is higher than at the 180° side; positive potential (blue) shows a reverse distribution, highest density being at the 180° side. At physiological ionic strength (Figure 1, top right), preferential distribution of the positive potential (180° side) is more evident, whereas higher density in negative potential (0° side) is less evident, because most Coulombic interactions are masked by counter-ions. When considering individual stem variation, net charge roughly doubles from the −8 *e* value of H1 and H9 to −15 *e* of H7. However, similar net charge does not necessarily correspond to similar distribution (along the stem) of the potential, that can preferentially locate at either the VED-distal stem subregion (left side in figure) or at the VED-proximal one (right side). This is the case for H1 and H9 stem, sharing net charge −8 *e*, and showing (more evident at I = 0 mM) preferential VED-distal and VED-proximal negative potential, respectively. Such preferential VED-distal location of the negative potential shown by H1 is conserved also in the other two stems from Group 1, in spite of their different net charge (−10 *e*). Positive potential is more homogeneously distributed along all stems. Heat maps and corresponding density plots (Figure 1, bottom) depict the overall similarity among HA stem electrostatic profiles. Comparison between the density plots at I = 0 mM and I = 150 mM highlights a general increase in distance, i.e. a peak shift from middle ED (green region) to high ED (cyan/blue region). When comparing Group 1 stems to those from other groups it can be noticed that - at both ionic concentrations - H3 is slightly closer to Group 1 than H7, while H9 is far apart. However, H9 distance is not homogeneous with respect to the three Group 1 stems, as it is closer to H2 than to H1 and H5. Indeed, H9 stem is also quite far from H7 because it shows the highest overall distance, with respect to other stem structures. When using WebPIPSA, the distance matrix of the electrostatic potential can also be displayed as a tree referred to as ‘epogram’ (electrostatic potential diagram). Epograms [see Additional file 2] further highlight at both ionic concentrations that: (i) H9 stem shows unique electrostatic features (i.e., the highest ED with respect to other stems) and (ii) H7 is closer to H3 than to other stems. This clustering is confirmed when using Carbo SI. The highest electrostatic distance shown by H9 might depend on its mammalian (swine) rather than avian origin. Therefore, structural models were obtained by homology modeling for avian H9 (A/Chicken/Jiangsu/H9/2010(H9N2), UniProtKb AC: G8IKB3) and horse H3 (A/Equine/Mongolia/56/2011(H3N8); UniProtKb AC: J9TJ60), using as structural templates 1JSD (H9) and 1MQL (H3), respectively and investigated using WebPIPSA. Comparison of epograms alternatively including either the avian H9 model or the swine template showed conservation of the highest distance observed for H9: at I = 0 mM, swine/avian epogram clustering was congruent; at I = 150 mM, avian H9 sorted with H3 and H7; this notwithstanding, highest distance of H9 from other HAs was anyway kept [see Additional file 3]. Concerning equine H3, it sorted like avian H3 at both I = 0 mM and I = 150 mM (congruent epograms see Additional file 3). In conclusion, electrostatic distance is not significantly influenced by taxonomy hence segregation depends on HA-specific features.

#### RBD subregions

As with the stem subregion, charge separation onto the RBD surface is more evident at I = 0 mM. Group 1 RBDs have an overall slightly negative (H1 and H2) or neutral (H5) net charge, which is positive (up to +3*e* in H3) in other groups. At large, the RBD net charge is less negative than stems (Figure 2, top). Side disposition in RBDs is not ‘side preferential’ as for stems, and no meaningful difference is observed when comparing the 0° and 180° views. However, preferential local distribution is clearly apparent also for RBDs, when a roughly orthogonal axis is considered: negative charges are densely distributed at the VED-proximal region (left side in figure), whereas charge of the VED-distal region (right side) is more positive. This is particularly evident for Group 1 RBDs at I = 0 mM. At physiological ionic strength, such preferential distribution is less evident, in particular for H3, where differently charged patches are interspersed. Peaks at the blue/purple regions in density plots (Figure 2, bottom) depict high electrostatic distances at both ionic strengths. Surprisingly - and independently on using either Hodgkin or Carbo SI - at I = 150 mM, the electrostatic potential of the H5 RBD is closer to H9 and H7 than to RBDs from H2, in spite H5 and H2 belong to the same Group. Splitting of Group 1 is confirmed by epogram [see Additional file 2] at I = 150 mM: H5 and H1 create a new cluster with H7 and H9.

#### HA1 subregions

Once the electrostatic analysis is repeated for the whole HA1 region, including the VED and F’ subregions in addition to the RBD [14], the most evident difference is an overall shift towards net positive charge (see upper panels in Figures 2 and 3), according to the presence of basic patches in F’ subregions [2,6]. Comparison of density plots (RBD vs. HA1) shows that peaks similarly locate at the high distance blue/purple regions (see lower panels in Figures 2 and 3) but, at I = 150 mM, Group 1 no longer splits, as H1, H2 and H5 form a cluster including H9. Resembling RBD distances, it also occurs with HA1 that members from Group 1 (H1 and H5) can be closer to an outgroup (H9) than to a member of the same group (H2) (see at I = 150 mM both heat map in Figure 3 and epogram in Additional file 2). This parameter independent evidence further highlights the relevance of counter-ions to shape the final electrostatic profile, as well as the possible disagreement between classic clustering (based on phylogenetic and serologic data) and electrostatics of the RBDs.

#### Monomers

The net charge is negative for all monomers, ranging -4*e* to -11*e* (Figure 4, top). Evidence that the net charge is quite negative for all stems (−8*e* to -15*e*) while being close to 0 for RBDs (−1*e* to +3*e*), stresses the total charge balancing by local basic patches in VED and F’ subregions. Once again, peculiar electrostatic features are evident (and SI independent) for H9, characterized by the less negative net charge and forming its own branch at both I = 0 mM and I = 150 mM (heat maps in Figure 4, bottom, and epograms in Additional file 2). Disagreement with serological and phylogenetic data is less evident when performing electrostatic analysis with entire monomer structures, as shown by clustering of Group 1 members in Figure 4 and Additional file 2.

#### Trimers

Once the entire haemagglutinin functional unit is analyzed, disagreement with serological and phylogenetic clustering is highlighted again by Group 1 splitting; in particular (and independently on which SI is used) at I = 0 mM, H1 sorts separately from H2 and H5 (see Figure 5, trimer heat maps and Additional file 2, trimer epograms). Such splitting is also observed at I = 150 mM, as H5 and H1 sort with H9 and H7, whereas H2 sorts out with H3. Comparison of net charges from monomers and corresponding trimers unveils striking doubling vs. triplication mechanisms: trimer net charge values for H1 and H3 is roughly three-fold with respect to corresponding monomers, or even more (−37*e* vs. -11*e*) for H5. Instead, trimer values are only roughly twofold increased for H2, H7 and H9. Therefore, different orientations of monomers within corresponding trimers results in significant modulation of the trimer surface electrostatic charge and this in turn can be quite relevant to HA interactions. Different HA clustering at I = 0 mM and I = 150 mM may highlight the importance of ionic screening of coulombic interactions [31,32]. As a final remark, based on absence of net charge-based clustering in any executed electrostatic analyses, the spatial distribution of electrostatic potential is suggested to be more suitable than net charge alone for eventual use as a further ‘signature’ for protein/domain function.

### Hydrophobicity analysis

Search for HA-specific motifs/signatures can be integrated by hydropathy analysis. Both electrostatics and hydrophobicity are key determinants in surface properties hence in regulating protein interactions. In particular, hydrophobic patches located at the protein surface create unstable areas. The identification of well-defined patches rather than a ‘patchwork surface’ of hydrophobic and hydrophilic areas can thus shed light on molecular evolution of haemagglutinin. Stem, RBD and HA1 profiles were obtained and compared using ProtScale [33] and Protein Hydrophobicity Plots [34]. Profiles from the stem subregions did not unveil any clearly meaningful difference and thus are not shown here.

#### RBD subregions

**Figure 6:**
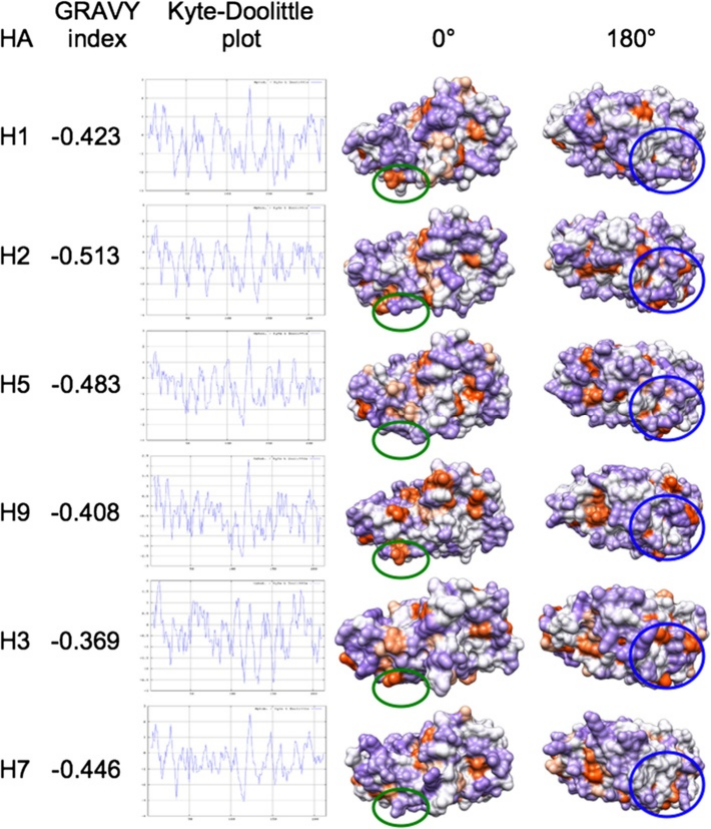
**Hydrophobicity analysis of the RBD subregions from the six available HA structures.** GRAVY Index, Kyte-Doolittle plots and surface hydrophobic (orange) and hydrophilic (violet) patches (as both 0° and 180° views) are depicted.

Figure 6 shows GRand AVerage hYdrophobicity (GRAVY) indexes, Kyte-Doolittle plots and 0° +180° surface hydropathy views for the RBDs from the six available HA structures. Similar to total electrostatic charges, GRAVY indexes are reported here for completeness of information; however, they are not suitable for use as evolutionary or functional fingerprint. In fact, variation of GRAVY values amongst the six RBDs does not correspond to high conservation and fine tuning of their surface patches as depicted in 0° and 180° views. However, comparison of Kyte-Doolittle plots could infer variation at specific positions. Plots in Figure 6 always start by residue 1 because the default numbering system from the software refers to analyzed sequence fragments (RBDs in this case); therefore, for Reader’s convenience, hereafter we report both real numbers (referring to complete protein sequences) and software output numbers (between parentheses). Within Group 1, the highest intra-group hydrophilicity is shown by H1 positions Arg223 (160) of the 220-loop and by H2 at positions Asn80, Ser136 and Glu202 (17, 73 and 139). At position 112 (49), H1 is significantly more hydrophobic (Ile) than H2 and H5 (Asn). Inter-group comparison highlights in H3 three hydrophilic peaks centered on residues Asp191, Thr208 and Gln227 (114, 135 and 154), as well as increased hydrophobicity of H7 in subregion 105–155 (50–100). Comparative analysis of surface patches unveiled possible HA-specific fingerprints. Within Group 1, variation concerns both the VED and RBD subregions. Such variation is even more evident when extending comparison to H9, H3 and H7. Hydrophobic patches (light and dark orange) are variable in terms of position and area. Comparison of 0° views highlights a large orange surface encompassing the VED-RBD border, specific to H9. Moreover, H5 and H7 show at the VED subregion a hydrophilic (violet) surface (green ovals) that in other HAs includes at least one small orange patch. Comparison of 0° views shows that H2 and H3 share three hydrophobic spots in an RBD subregion (blue circles) where other HAs can lack one, two or even all such spots. Further variation can be observed, and in general it seems to concern ‘position-shifting’ rather than significant difference in the total ratio of hydrophilic/hydrophobic surfaces. Therefore, combined variation in both electrostatic and hydropathy features is likely to fine tune local interaction properties of the different HA RBDs.

#### HA1 subregions

Apart from differences already observed in the RBD subregion, no further meaningful variation was found among HA1 hydropathy profiles. The only relevant evidence concerns the hydrophilicity peak at position 297 in H3 haemagglutinin (not shown).

### Structural modeling of H5N1 clades and electrostatic features comparison

**Figure 7:**
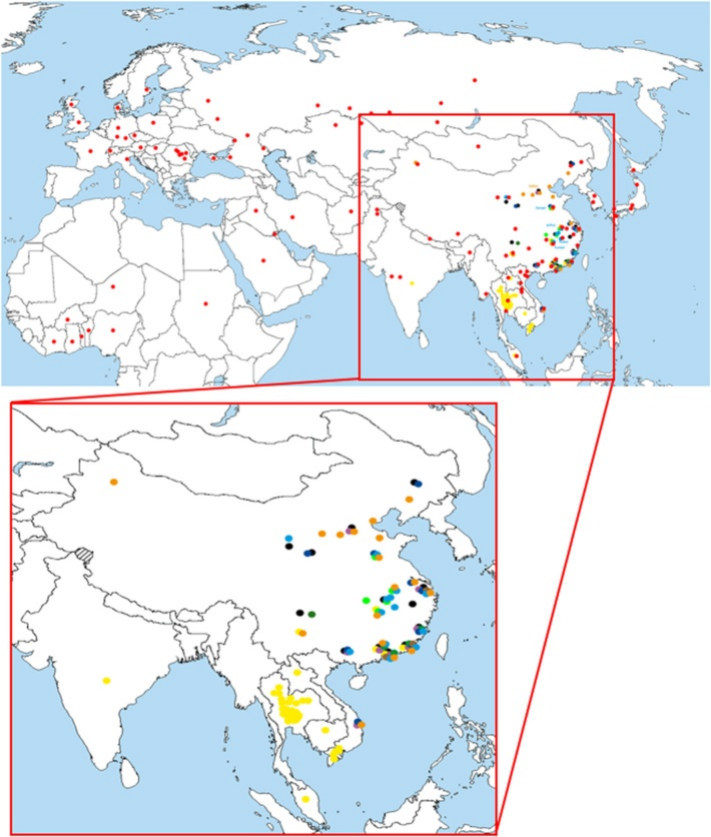
**Geographical spread of H5N1 clades.** Outbreak areas for each clade are color coded as follows: 0, black; 1, yellow; 2, red; 3, violet; 4, dark green; 5, dark blue; 6, light green; 7, orange; 8, brown; 9, cyan.

Electrostatic features can vary among different types of haemagglutinins (see above). This prompted us to further investigate on differential electrostatic features as a possible fingerprint for monitoring viral evolution, i.e. as a tool to distinguish among circulating/spreading and extinguished H5N1 clades. Table 2 resumes relevant data concerning the ten clades used for this analysis; their geographical spread is shown in Figure 7. Spreading of no longer circulating clades (0, 3, 4, 5, 6, 8 and 9) is restricted to the eastern part of China and to Vietnam (see Figure 7, zoom in map); noticeably, all such clades share one or more outbreak areas with the most ancient clade (clade 0, black spots). Among circulating clades, clade 7 was also found in western China and clade 1 also spread towards India and Indochina countries (Thailand, Laos, Cambodia and Malaysia). The widest spreading concerns circulating clade 2 (red dots in the upper map of Figure 7), having reached Japan and Korea, Mongolia, Russia, several countries from Middle-East and Europe (including UK) as well as a number of African countries from the Northern hemisphere. So far, spreading of H5N1 viruses neither concerns Americas nor any country from the Southern hemisphere (Oceania and sub-equatorial Africa).

**Table 2:** **H5N1 clades**

**Clade**	**Year**	**Strain name**	**Genomic Ac**	**Protein Ac**
0	1996-2002	A/Goose/Guangdong/1/1996	AF144305.1	AAD51927.1
1 (c)	2002-2003	A/Quail/Shantou/3054/2002	CY028946.1	ACA47648.1
2 (c)	2005	A/Bar-headed Gooze/Qinghai/75/2005	DQ095619.1	AAZ16276.1
3	2000-2001	A/Duck/Hong Kong/2986.1/2000	AY059481.1	AAL31387.1
4	2002-2003	A/Duck/Shantou/700/2002	CY028943.1	ACA47615.1
	2005-2006			
5	2000-2003	A/Duck/Zhejiang/52/2000	AY585377.1	AAT12042.1
	2004			
6	2002-2004	A/Duck/Hubei/wg/2002	DQ997094.1	ABI94747.1
7 (c)	2002-2004	A/Chicken/Shanxi/2/2006	DQ914814.3	ABK34764.2
	2005-2006			
8	2001-2004	A/Chicken/Hong Kong/61.9/2002	AY575876.1	AAT39076.1
9	2003-2005	A/Duck/Guangxi/50/2001	AY585375.1	AAT12040.1

Periods (years) of circulation, strain names (based on year and location of identification) and accession numbers (for both genomic and protein data) are reported for each clade. Circulating clades are marked by (c).

Based on a very high, average % identity (over 90%) of the clade target sequences with the available structural H5 template (PDB: 3S11), structural models for clades 0 to 9 were obtained by homology. Given that distribution of surface charge is strongly influenced by the orientation of side chains, models refinement was performed using a number of tools based on different algorithms: SCWRL [35,36], ModRefiner [37] and SCit [38]. Then, QMEAN server was used to check model quality; QMEAN is a scoring function that measures multiple geometrical aspects of protein structure, ranging 0 to 1 with higher values indicating more reliable models [39]. QMEAN scores for each refined or not refined model (mQMEAN) and the average QMEAN score for each ten clades model series (aQMEAN) was calculated. Models refined by SCWRL showed the highest aQMEAN (0.734), with highest mQMEAN for clades 0, 1, 2, 3 and 5. However, quality was similarly good when models were not refined (aQMEAN: 0.724; highest mQMEAN for clades 6 and 7) or refined by ModRefiner (aQMEAN: 0.720; highest mQMEAN for clades 4, 8 and 9), confirming once again reliability and robustness of the SWISS-MODEL homology modeling method [40]. SCit refined models showed the lowest average quality (aQMEAN: 0.702). Therefore, electrostatic analyses were performed thrice, using the ten clades models: (i) refined by SCWRL, (ii) refined by ModRefiner and (iii) not refined.

Preliminary comparison at trimer and monomer level showed meaningful variation only at the VED-RBD subregion. In fact, direct comparison of stems did not allow for inferring any clade-specific signature as all clades were found to share - at both I = 0 mM and I = 150 mM - the typical isocontour of the H5 stem (see Figure 1, top). Moreover, apart from electrostatic differences in the VED-RBD subregion, no further meaningful variation was observed among HA1 isocontours. This prompted us to ‘zooming in’ variation analysis at the RBD subregion level.

**Figure 8:**
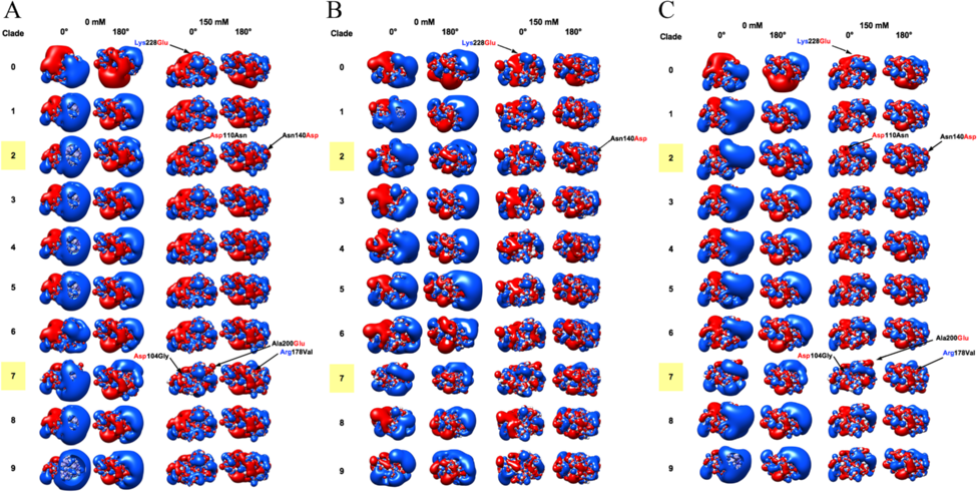
**Isopotential contours of the RBD subregions from H5N1 clades 0 to 9.** See Figure 1 caption for color code and definitions. Isopotential contours are plotted at ±1k_B_T/e. Specific mutations discussed in the text are highlighted. Positively charged residues are written in blue and negatively charged ones in red. Panels: models refined using SCWRL **(A)**, ModRefiner **(B)** or not refined **(C)**.

**Figure 9:**
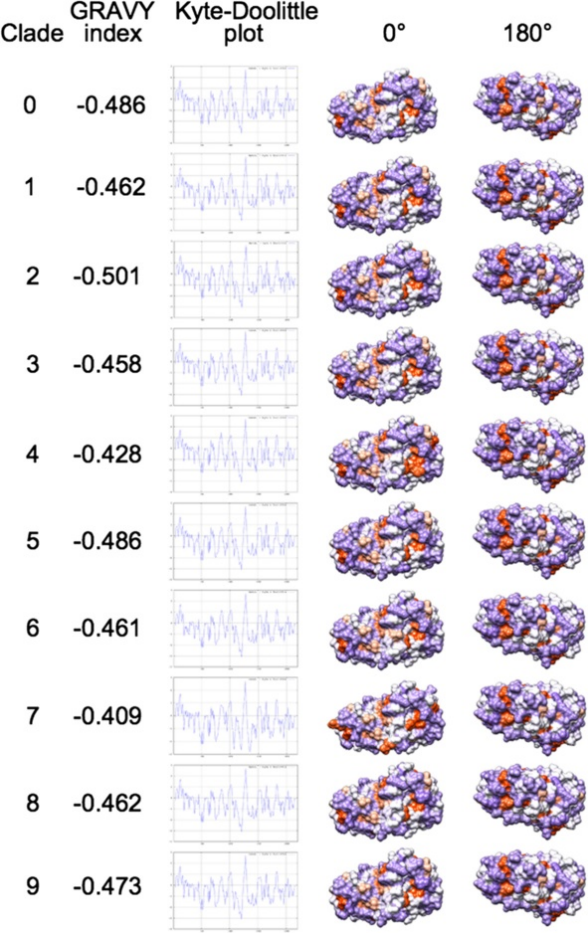
**Hydrophobicity analysis of the RBD subregions from H5N1 clades 0 to 9.** See Figure 6 caption for color code and definitions.

Figure 8 illustrates local charge variation in RBD isocontours among H5N1 clades. Even though variation is more evident at I = 0 mM, meaningful difference is kept hence highlighted at physiological ionic strength. It is noteworthy that, independently on models are refined or not and on algorithm used for refinement, the same relevant local changes in RBD isopotential contours are found (see Figure 8, panels A to C). Early clades evolution is characterized by a charge shift event at the 220-loop: in the most ancient clade (clade 0), the side chain of amino acid 228 shows either negative (Glu: 50/89 and Asp: 1/89 sequences) or positive (Lys: 38/89 sequences) charge. The positive charge is ‘fixed’ in the most recent, and still circulating clades 2 (Lys: 308/310, Glu or Asp: 0/310 sequences) and 7 (Lys: 25/26; Glu: 1/26 sequences) (see Figure 8 and Table 3). Further loss of a negative residue (Asp) concerns the VED isocontour at the 110-helix region. Table 3 shows that in clade 0, position 110 is negatively charged (Glu or Asp: 67/89 sequences) or polar, non-charged (Asn: 22/89 sequences). This negative charge is almost completely lost in clade 2 (Asp: 3/310, Glu: 0/310), while being retained (Asp: 26/26) in clade 7; however, this latter clade shows ongoing loss of the negative charge at position 104 (Asp: 15/26; Gly: 11/26), that is positively charged in 100% of clade 0 and clade 2 sequences (Figure 8 and Table 3). In clades 2 and 7, such ‘denegativization’ of the VED isocontour is somehow counterbalanced by negativization (or depositivization) at the properly receptorial part of the RBD. In clade 2, this depends on Asn140Asp mutation (in 307/310 sequences) while in clade 7 both depositivization (Arg178Val in 8/26 sequences) and negativization (Ala200Glu in 12/26 sequences) mutations are observed (Figure 8 and Table 3). Intriguingly, when considering aforementioned replacements altogether, evolution of H5N1 still circulating clades seems having been characterized by an isocontour rearrangement based on a VED-to-RBD flow of negative charges; this process is ‘partial’ hence seemingly in progress in clade 7 (mutation arose in the clade and it is present, at least so far, in less than 50% sequences) whereas it is complete and ‘fixed’ (99% sequences) in clade 2. Given that comparison of the six different HA structures identified HA-specific variation in both electrostatic and hydropathy features, and that specific electrostatic signatures of the RBD could also be associated to the ten H5N1 clades, clades analysis was integrated by comparison of the RBD surface hydropathy profiles (Figure 9). As for electrostatic analysis, the most ancient clade (clade 0) is the reference for tracking hydropathy profile variation along clades evolution. As previously explained, hereafter both real protein sequence numbering and (between parentheses) software output numbering is reported for Reader’s convenience. Clade 3 shows no substantial difference with respect to clade 0, at least in terms of hydropathy plots. Instead, clade 4 shows increased hydrophilicity at position Asn211 (148). Clade 1 shows increased hydrophobicity around position Ser140 (77). Replacement at position 124 of a polar residue in clade 0 by Ile in all other clades results in increased hydrophobicity. Intriguingly, the hydropathy profile of clade 7 resembles the one of H3 haemagglutinin, including its aforementioned three hydrophilicity peaks. Please note that the apparent disagreement among positions of the three H3 peaks in Figure 6 and those from Clade 7 in Figure 9 is not confirmed in real numbering, as plot shift is determined by ten extra residues present in the really N-terminal region of H3. Apart from difference illustrated so far for the RBD, no further meaningful variation was observed when comparing other HA1 subregions or the stem profiles (not shown).

**Table 3:** **Mutations in H5N1 clades 0, 2 and 7**

**Clade**	**Sequences**	**Position**					
		**104**	**110**	**140**	**178**	**200**	**228**
0	89	Asp = 89	Asp = 64	Asn = 86	Arg = 89	Ala = 89	Glu = 50
			Asn = 22	Asp = 3			Lys = 38
			Glu = 1				Asp = 1
2.2	310	Asp = 310	Asn = 302	Asp = 307	Arg = 284	Ala = 307	Lys = 308
			Lys = 4	Asn = 2	Ile = 26	Gly = 3	Asn = 1
			Asp = 3	Gly = 1			Gln = 1
			Ser = 1				
7	26	Asp = 15	Asp = 26	Asn = 24	Arg = 16	Ala = 14	Lys = 25
		Gly = 11		Asp = 2	Val = 8	Glu = 12	Glu = 1
					Gly = 2		

For each clade, the number of analyzed available sequence is shown. For each position (numbering refers to clade 0 sequence), the type of present residues and corresponding number of sequences showing that residue is shown.

## Conclusions

Evidence from this work shows that sequence homology is often, but not always, related to structural similarity and vice versa. In fact, in some instances, protein domains with less related sequences can show intriguing structural closeness. Therefore, in order to obtain a more complete view of the ‘functional evolution’, phylogenetic analyses based on sequence comparison and resulting in trees, might be integrated taking into account information from structural comparison. Dissimilarity in secondary structure elements does not always result in different antigenic properties. Sometimes, secondary structure is not prominent to the molecule antigenicity. Indeed, electrostatic features are crucial to interactions and in fact electrostatic profiles of the RBD subregion varies amongst different HAs. On the other hand, stems, HA1, monomers and trimers topology appears to be variable. As shown by H9 and H3 modeled structures, electrostatic profiles seem to depend on HA type rather than organism source. Hydrophobicity analysis reveals that local, ‘spot’ variation especially concerns the RBD subregion. No flow of hydrophobicity/hydrophilicity is observed as for charge flow in the electrostatic analysis. In H5N1 clades comparison, from an electrostatic point of view, meaningful variation concerns only the VED-RBD subregion. Intriguingly, a charge flow specifically concerns still circulating clades 2 and 7, where ‘denegativization’ of the VED isocontour is counterbalanced by negativization in the RBD. It is noteworthy (and a ‘positive mark’ for robustness of the observation) that the same specific differences are found when comparing refined or not refined clade models or models refined using different algorithmic strategies (as SCWRL is rotamer library-based [35,36] while ModRefiner is based on two-step atomic-level energy minimization [37]). Given that local charge concentration is typical for antigenic epitopes, it is tempting to speculate that charge redistribution in such clades might have contributed to antigenic escape hence to their evolutionary success and spreading. Indeed, such an hypothesis is in agreement with evidence that charge redistribution on the RBD characterizes the two clades (2 and 7) which were able to spread over the largest geographical distribution and that, in particular, such redistribution is fixed in sequences from clade 2, which is the world most spread clade. It is noteworthy that also variation in hydrophobic patches is especially observed in the RBD subregion.

## Methods

### Structural templates and target sequences

The following structures from the Protein Data Bank (PDB) were used as templates for modeling: H1, PDB 1RUZ, from viral strain A/South Carolina/1/1918(H1N1); H2, PDB 2WR5, from Asian pandemic influenza virus of 1957; H3, PDB 1MQL, from viral strain A/duck/Ukraine/1963 (H3N8); H5, PDB 3S11, from viral strain A/Goose/Guangdong/1/1996 (H5N1); H7, PDB 1TI8, from viral strain A/turkey/Italy/214845/2002(H7N3); H9, PDB 1JSD, from viral strain A/swine/Hong Kong/9/98(H9N2). UniProtKb accession codes (AC) of target sequences modeled by H.M. and corresponding viral strains (VS) are the followings: H4, AC F2NZ53, VS A/duck/Guangxi/912/2008(H4N2); H6, AC H8PBW2, VS A/duck/Fujian/6159/2007(H6N6); H8, AC D4NQL7, VS A/northern pintail/Alaska/44420-106/2008(H8); H10, AC P12581, VS A/Chicken/Germany/n/1949 (H10N7); H11, AC D5LPX8, VS A/turkey/Almaty/535/2004(H11N9); H12, AC E6XYK2, VS A/mallard/Interior Alaska/9BM1907R1/2009(H12); H13, AC P13101, VS A/Gull/Astrakhan/227/1984 (H13N6); H14, AC P26136, VS A/Mallard/Astrakhan/263/1982 (H14N5); H15, AC Q82565, VS A/duck/Australia/341/1983(H15N8); H16, AC Q5DL23, VS A/black-headed gull/Sweden/3/99(H16N3). Given that original UniProtKb sequences indeed correspond to H0 precursors, sequence fragments missing in mature chains were manually removed to avoid improper structural alignment.

### Structural superpositions, Homology Modeling, model refinement and quality check

Structural superpositions were performed and viewed using UCSF Chimera [18] v. 1.8.1 (free download from [41]). Target protein sequences were modeled on best available structure templates using SWISS-MODEL [40]. Then, model structures were refined using SCWRL [35,36], ModRefiner [37] or SCit [38]. Model quality was checked via QMEAN server [39].

### Electrostatic surface analysis

Isopotential contours were calculated using UCSF Chimera 1.8.1: the software utility allows for connecting - through Opal web server - to the Adaptive Poisson-Boltzmann Solver (APBS) server [42]. Isopotential contours were then plotted at ±3k_B_T/e, ±2k_B_T/e and ±1k_B_T/e (RBDs). PDB2PQR was used to assign partial charges and van der Waals radii according to the PARSE force field [43]. Interior ε_p_ = 2 and ε_s_ = 78.5 were chosen for respectively the protein and the solvent [30,44,45], T = 298.15 K. Probe radius for dielectric surface and ion accessibility surface were set to be r = 1.4 Å and r = 2.0 Å, respectively. Electrostatic distance was calculated using the Hodgkin index and the Carbo index at the WebPIPSA server [46]. Rigid-body superposition was performed and electrostatic potential was computed using Chimera 1.8.1.

### Hydropathy analysis

Hydropathy analysis was performed using the Kyte-Doolittle scale implemented in Protein Hydrophobicity Plots [34] and in ProtScale at the ExPASy server [47,48]. In order to highlight hydrophilic regions likely exposed on the surface, a seven amino acids window was chosen; regions with score >0 are hydrophobic [33]. Hydrophobic/hydrophilic patches were plotted onto structures through Chimera 1.8.1.

## Additional files

Additional file 1:
**Two-pages figure relating HA stem secondary superstructures to immunogenic epitopes.**
Additional file 2:
**Multi-page figure reporting epograms for each analyzed HA subregions (stem, RBD, HA1) and for HA monomers and trimers.**
Additional file 3:
**Reports comparison amongst epograms for stem subregions obtained performing the WebPIPSA analyses with solved PDB structures or replacing either H9 or H3 templates by modeled structures.**

## Abbreviations

AC: Accession code
APBS: Adaptive PB Solver
ED: Electrostatic distance
Epogram: Electrostatic potential diagram
GRAVY: GRand AVerage hYdrophobicity
HA: Haemagglutinin
I: Ionic strength
N: Neuraminidase
PB: Poisson-Boltzmann
PDB: Protein data bank
PIPSA: Protein Interaction Property Similarity Analysis
RBD: Receptor-binding domain
RMSD: Root mean square deviation
SI: Similarity index
VED: Vestigial esterase domain
VS: Viral strain
WHO: World Health Organization
