# Supplementary material for: Comparative structural analysis of haemagglutinin proteins from type A influenza viruses: conserved and variable features
Source: BMC Bioinformatics. 2014 Dec 10;15(1):363. doi: 10.1186/s12859-014-0363-5 (PMC4265342; doi:10.1186/s12859-014-0363-5)
Supplement: Additional file 1: — Two-pages figure relating HA stem secondary superstructures to immunogenic epitopes. [file 12859_2014_363_MOESM1_ESM.pdf]

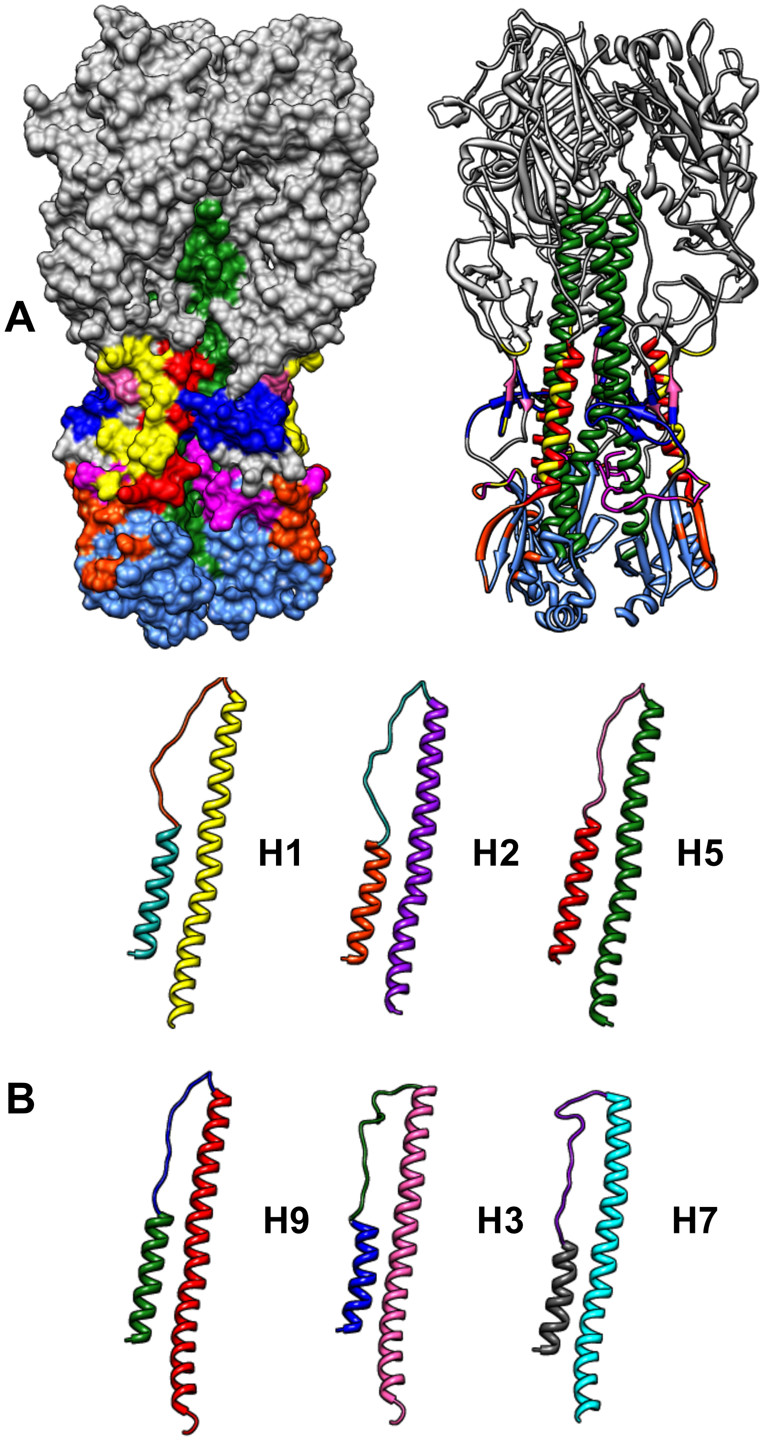
A
B
H1
H2
H5
H9
H3
H7

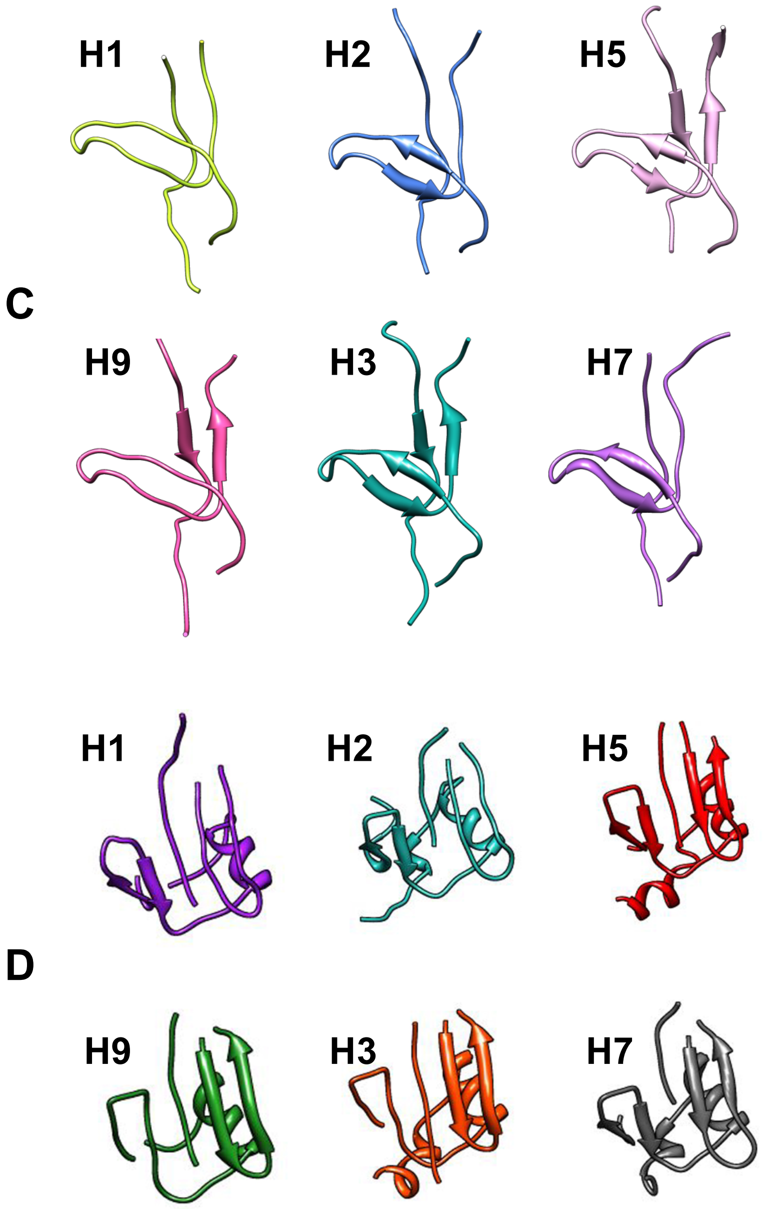

HA2 stem epitopes. Panel A: surface (left) and cartoon (right) representations of H5 trimers. Color code: RBD and VED, gray; A helix, red; C-D helices, green; fusion peptide, magenta; VED-proximal β region, blue; VED-distal β region, pale blue. Epitopes recognised by antibodies CR6261 and CR8020 are highlighted in yellow and orange, respectively. Panel B: comparison of the A-C-D α helices and B loop regions from the six available HA structures. Panels C and D focus, within the six available structures, on VED-proximal and VED-distal β regions, respectively.
