# Supplementary material for: Comparative structural analysis of haemagglutinin proteins from type A influenza viruses: conserved and variable features
Source: BMC Bioinformatics. 2014 Dec 10;15(1):363. doi: 10.1186/s12859-014-0363-5 (PMC4265342; doi:10.1186/s12859-014-0363-5)
Supplement: Additional file 2: — Multi-page figure reporting epograms for each analyzed HA subregions (stem, RBD, HA1) and for HA monomers and trimers. [file 12859_2014_363_MOESM2_ESM.pdf]

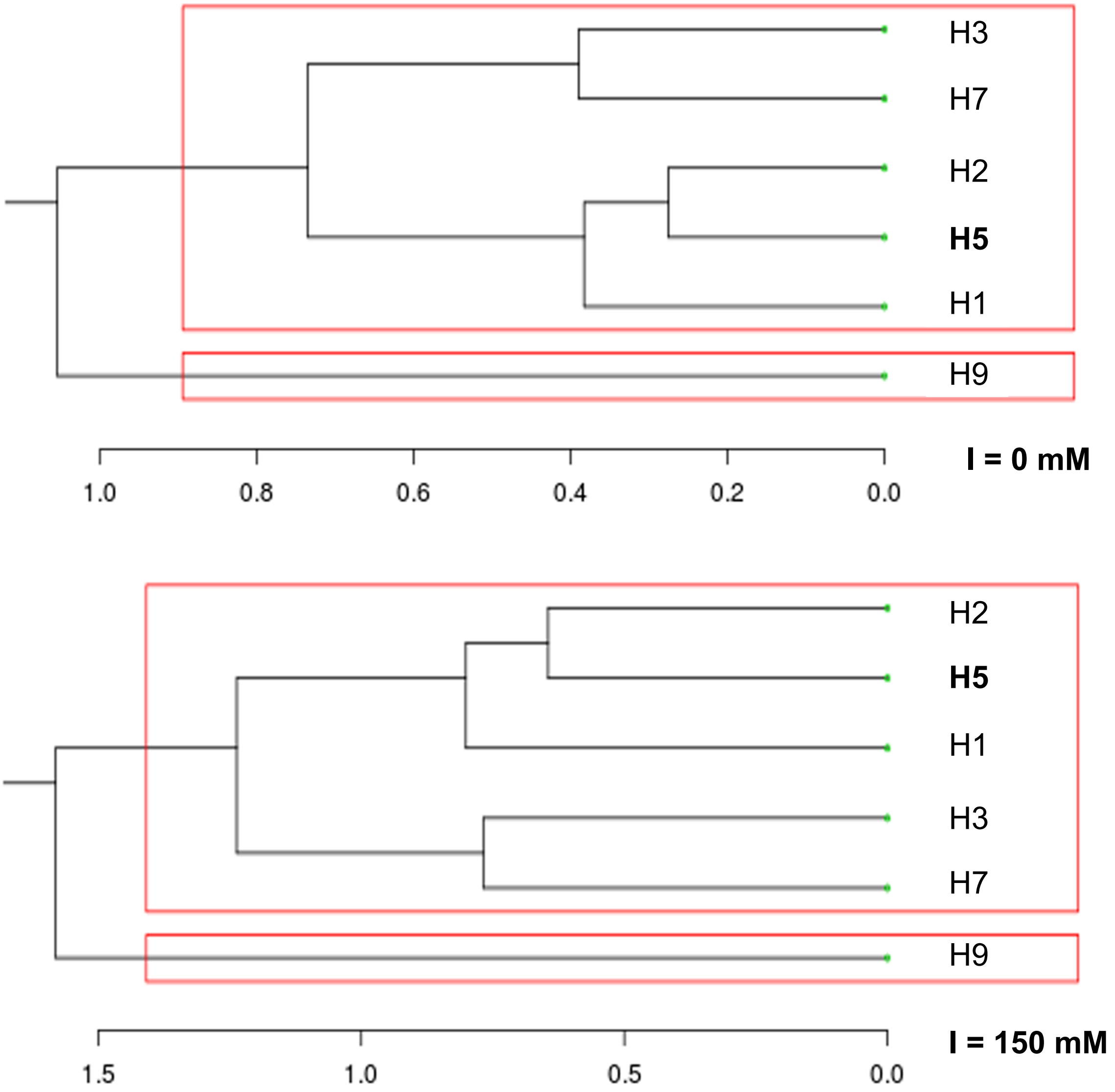

Epograms for the HA stem subregion. Epograms at I = 0 mM and I = 150 mM are shown. The horizontal axis of the epogram represents ED values.

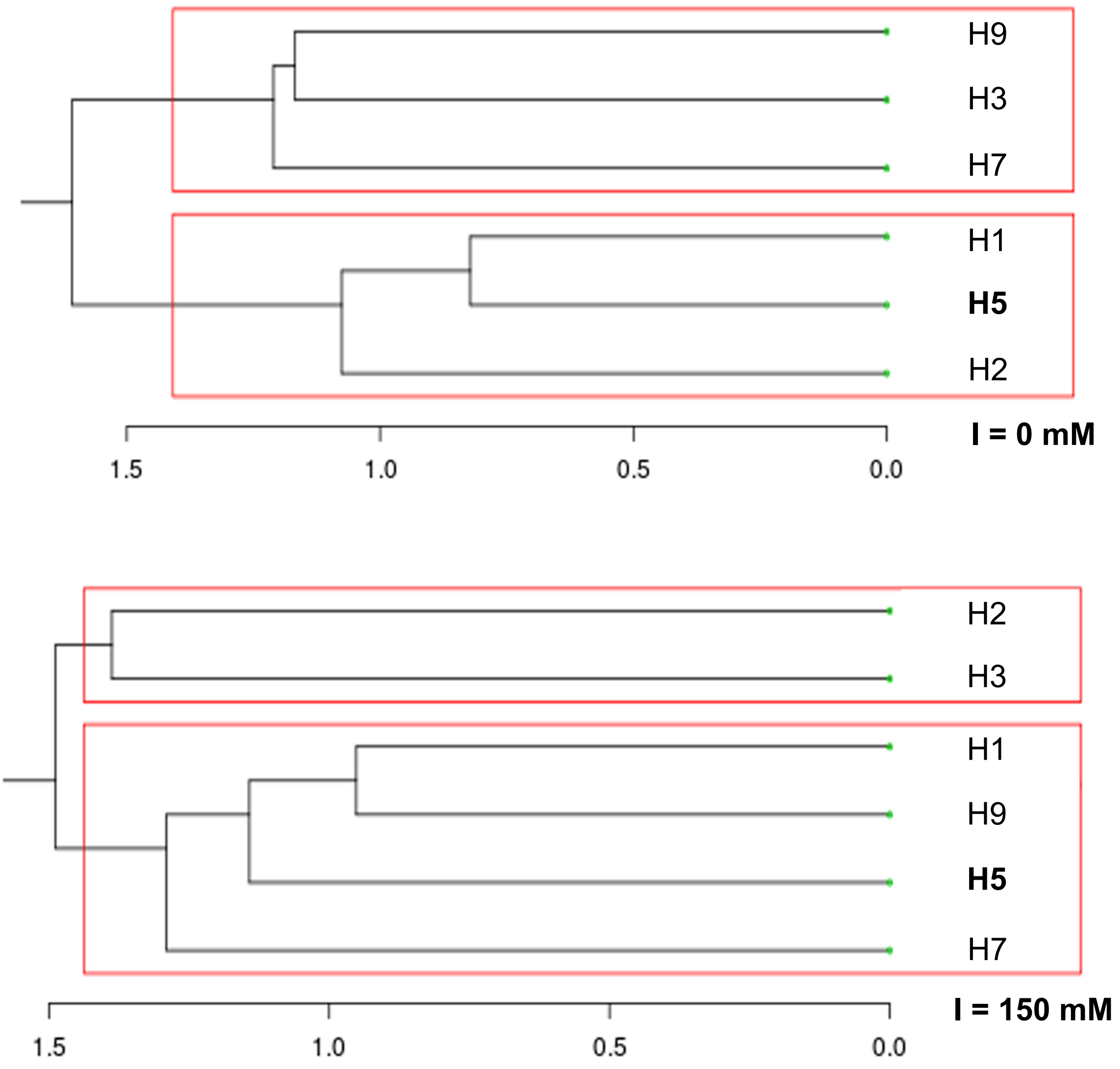

Epograms for the HA RBD subregion. Epograms at I = 0 mM and I = 150 mM are shown. The horizontal axis of the epogram represents ED values.

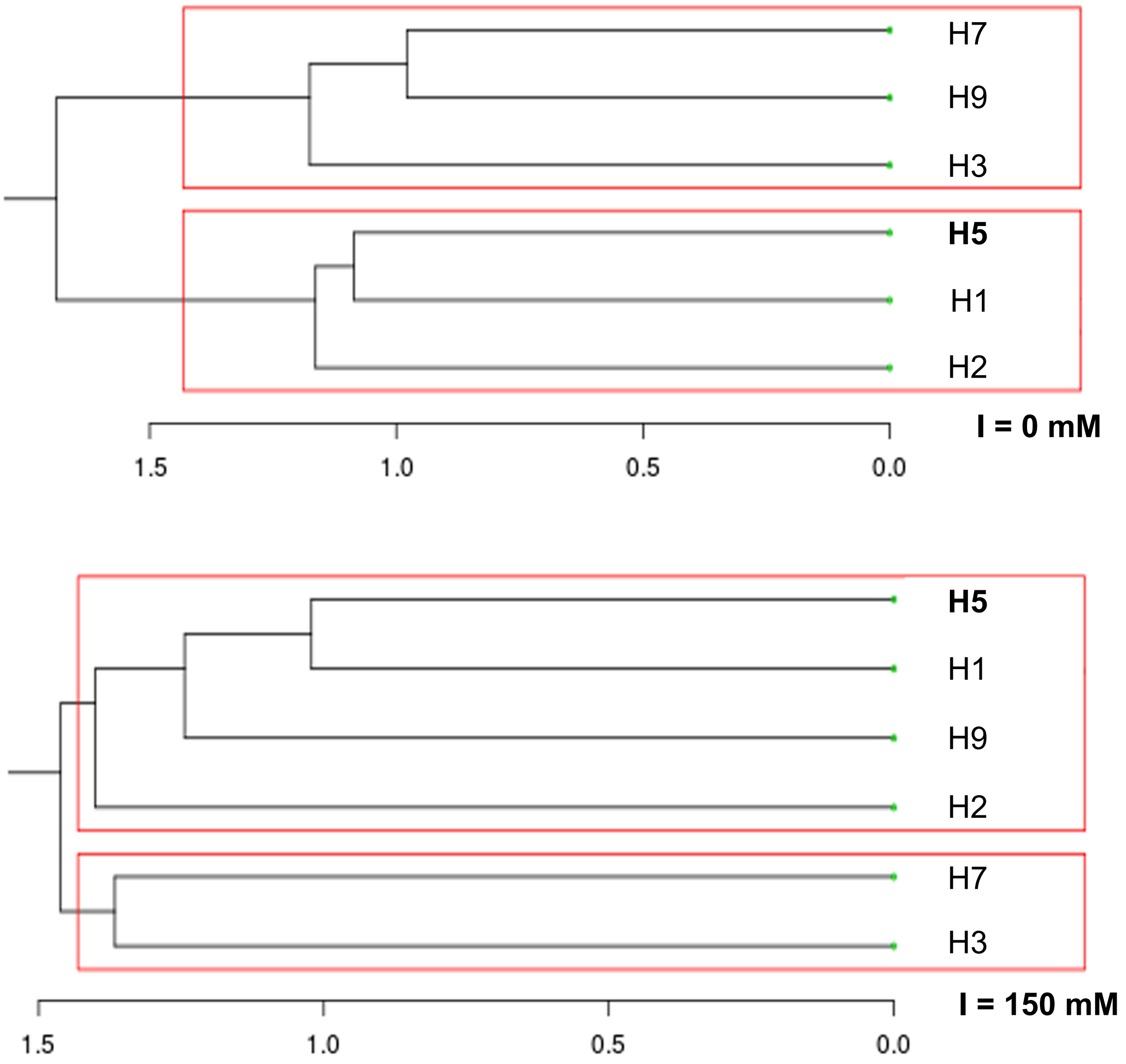

Epograms for the HA1 subregion. Epograms at I = 0 mM and I = 150 mM are shown. The horizontal axis of the epogram represents ED values.

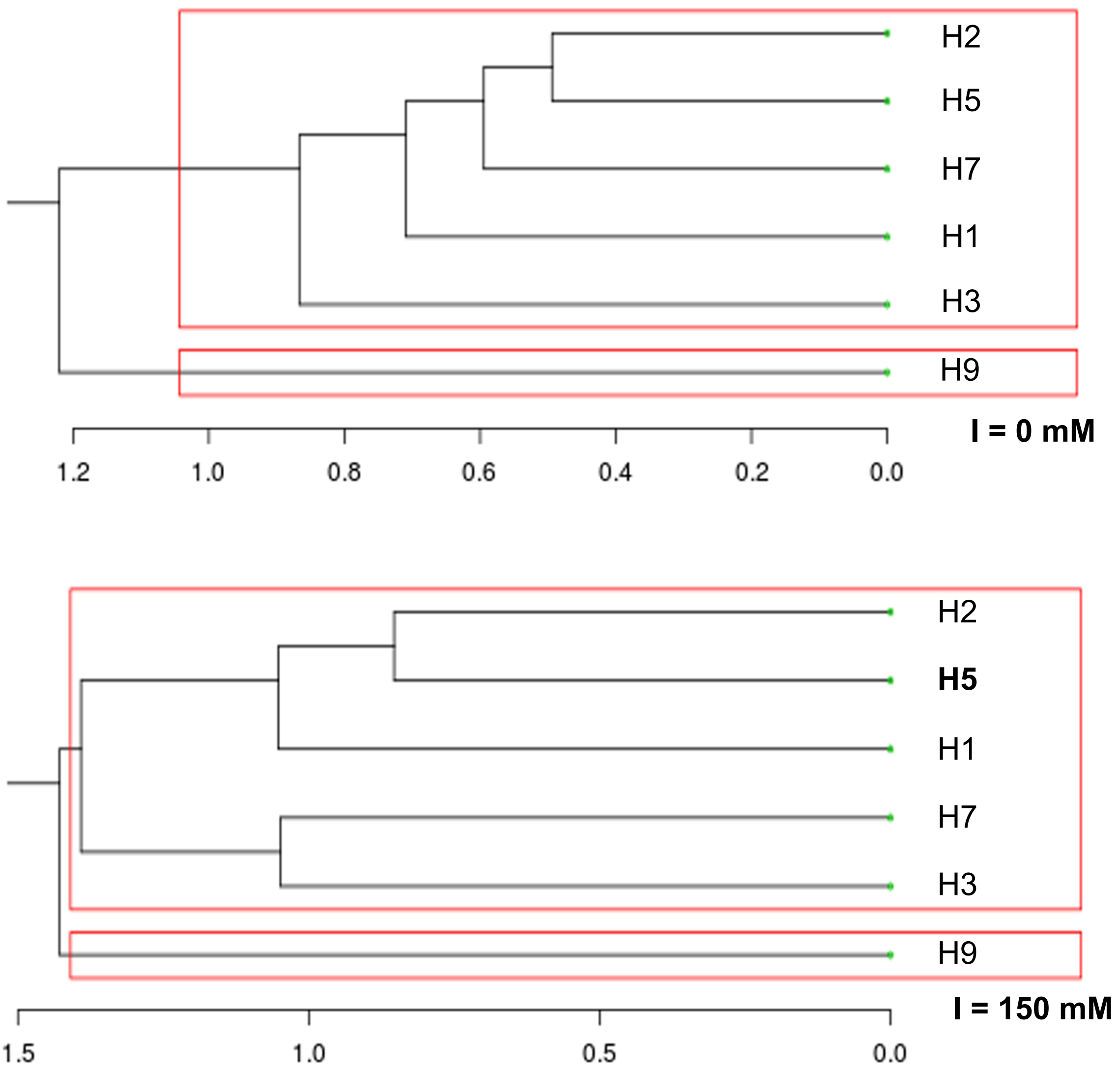

Epograms for the HA monomers. Epograms at I = 0 mM and I = 150 mM are shown. The horizontal axis of the epogram represents ED values.

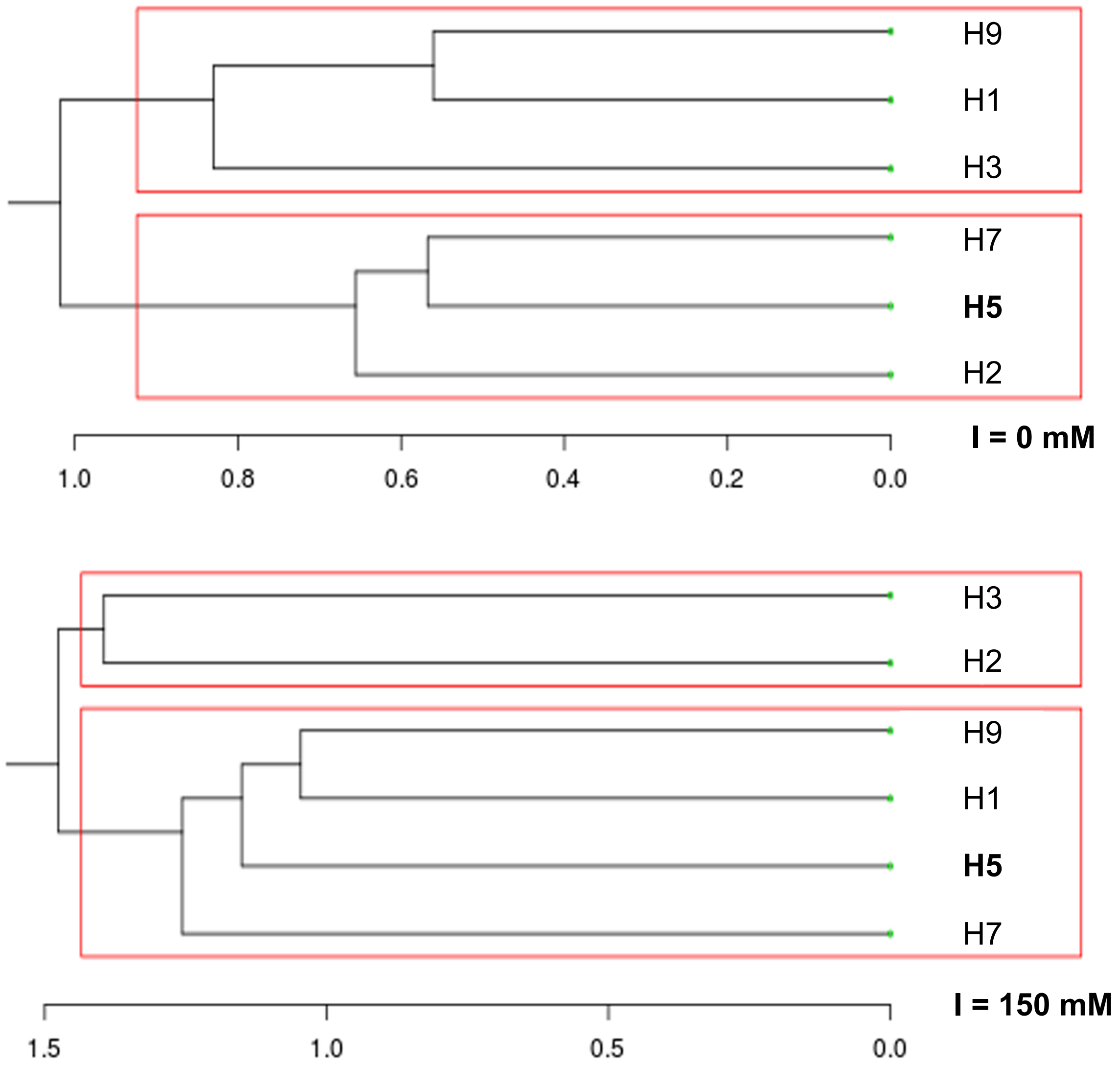

Epograms for the HA trimers. Epograms at I = 0 mM and I = 150 mM are shown. The horizontal axis of the epogram represents ED values.
