# Supplementary material for: Comparative structural analysis of haemagglutinin proteins from type A influenza viruses: conserved and variable features
Source: BMC Bioinformatics. 2014 Dec 10;15(1):363. doi: 10.1186/s12859-014-0363-5 (PMC4265342; doi:10.1186/s12859-014-0363-5)
Supplement: Additional file 3: — Reports comparison amongst epograms for stem subregions obtained performing the WebPIPSA analyses with solved PDB structures or replacing either H9 or H3 templates by modeled structures. [file 12859_2014_363_MOESM3_ESM.pdf]

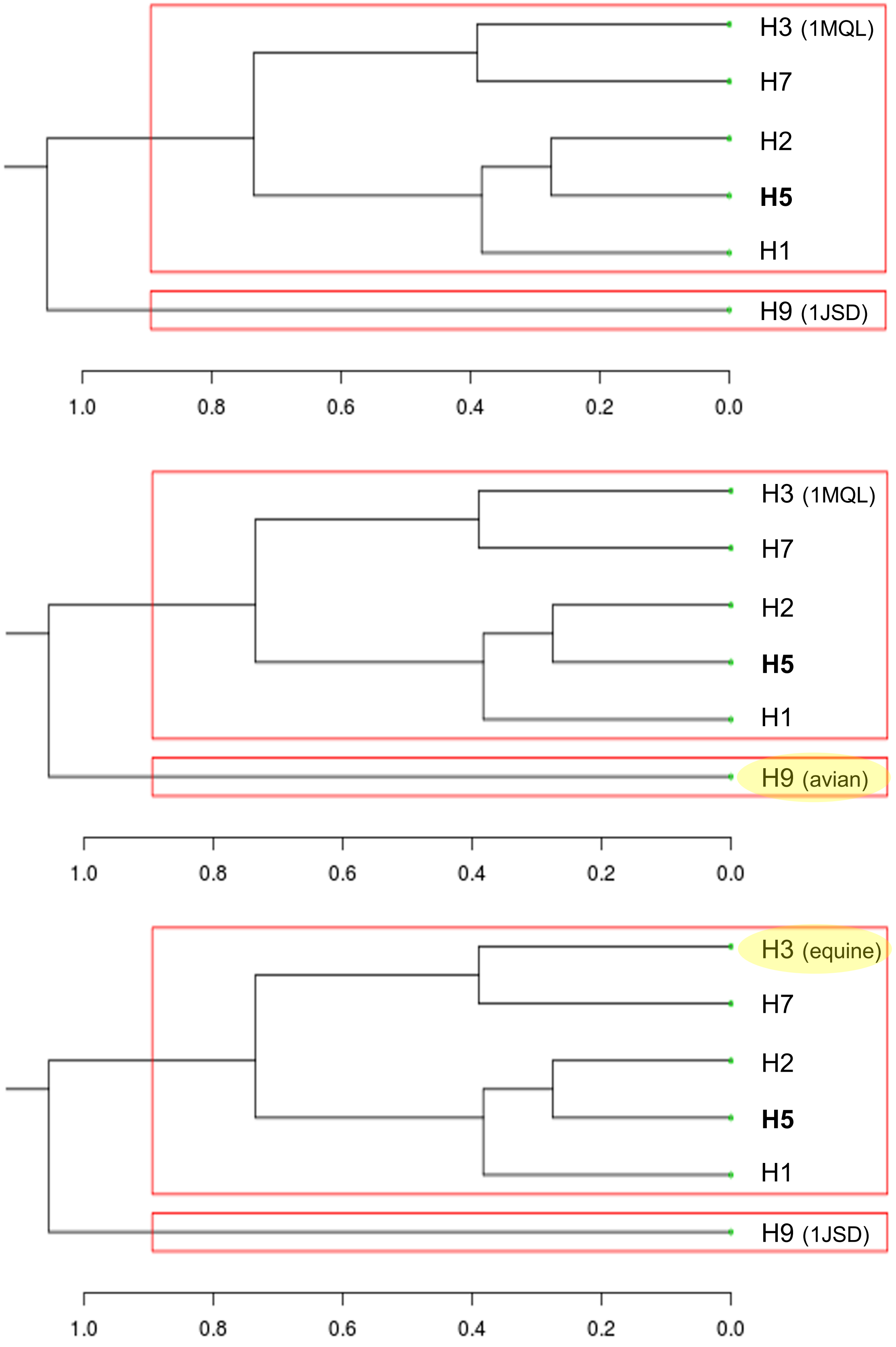

Epograms at I = 0 mM for the HA stem subregion. Modeled structures have yellow background. The horizontal axis of the epogram represents ED values.

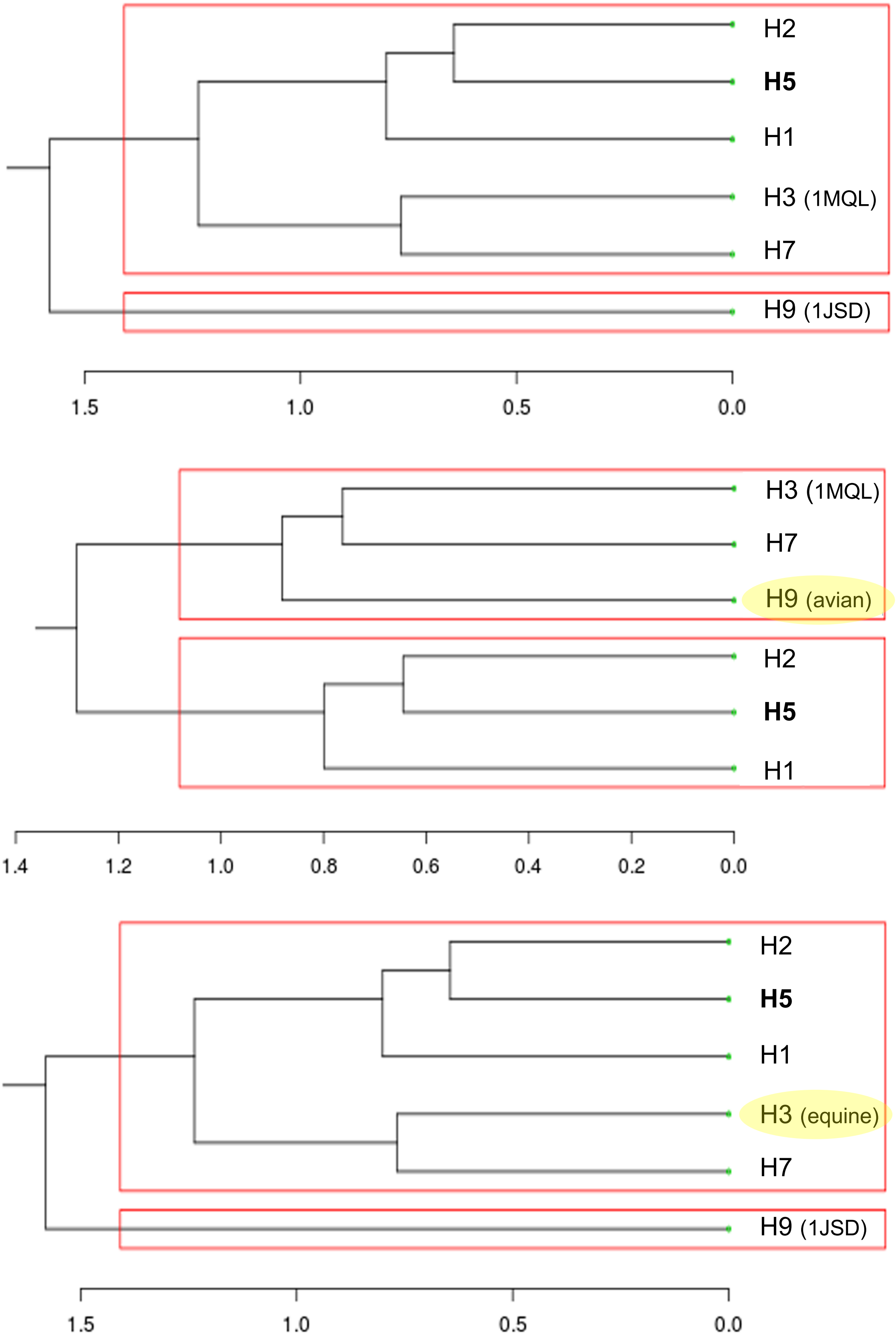

Epograms at I = 150 mM for the HA stem subregion. Modeled structures have yellow background. The horizontal axis of the epogram represents ED values.
